# 61. A Multidisciplinary Quality Improvement Initiative to Promote Penicillin Allergy Delabeling

**DOI:** 10.1093/ofid/ofab466.263

**Published:** 2021-12-04

**Authors:** Lea Monday, Jaclyn Michniak, Edward Zoratti, Allison J Weinmann

**Affiliations:** Henry Ford Health System, Detroit, Michigan

## Abstract

**Background:**

Penicillin (PCN) allergies are reported in up to 10% patients and are associated with adverse clinical and antimicrobial stewardship outcomes. Here we describe a multidisciplinary quality improvement (QI) initiative to facilitate PCN delabeling at a large urban hospital.

**Methods:**

Starting in August 2020, the departments of Allergy and Infectious Diseases (ID) began a joint QI effort to employ a part time allergist nurse practitioner (ANP) for PCN allergy assessment and delabeling. The ANP used a daily system generated list to identify and assess adult patients with PCN allergy and contact teams to request a consult. An ID fellow also assisted with identifying patients and contacting care teams. The ANP then offered skin/oral PCN challenge or direct label removal based on history after discussion with an allergist physician. Baseline, clinical, and allergy characteristics were compared between patients delabeled and not delabeled using Chi-square and Mann-Whitney U test. Primary endpoints were antibiotic utilization outcomes from index admission post ANP assessment to 30-days post discharge. Secondary endpoints included readmission, length of stay (LOS), mortality, and sustained removal of the PCN allergy at 30-days.

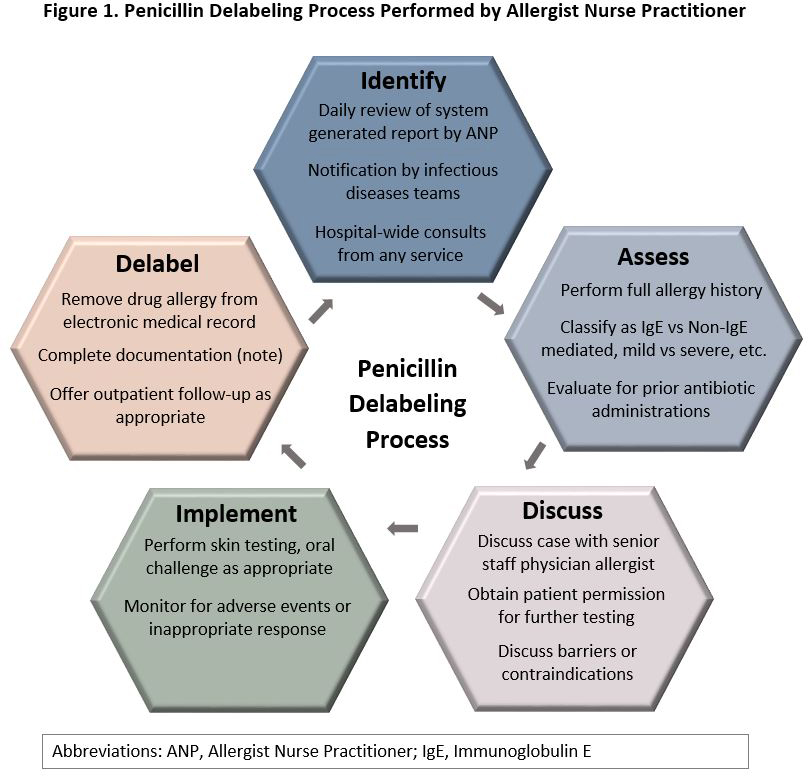

**Results:**

Between 30 August 2020 and 6 May 2021 (250 days), 139 PCN allergic patients were assessed (81 delabeled versus 58 not delabeled) (Figure 2). Some patients (37%) were delabeled via history alone, while 63% had further skin/oral testing. Baseline characteristics were similar between groups (Table 1). In the delabeled group, we observed increased narrow-spectrum PCN use (p< 0.001), and decreased vancomycin (p< 0.001), fluoroquinolone (p=0.013), carbapenem (p< 0.011), and overall restricted antimicrobial use (Table 2). Rates of 30-day readmission, LOS, and mortality were comparable. Four (5%) of delabeled patients had had PCN allergy re-entered in the chart at 30-days.

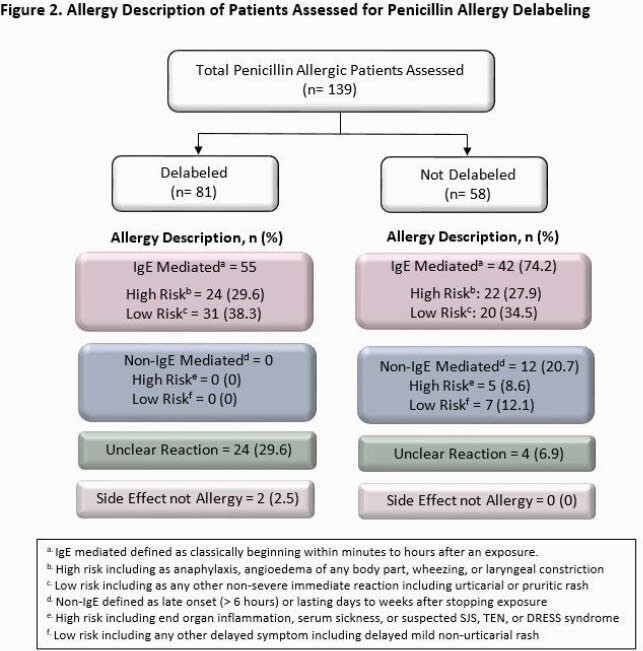

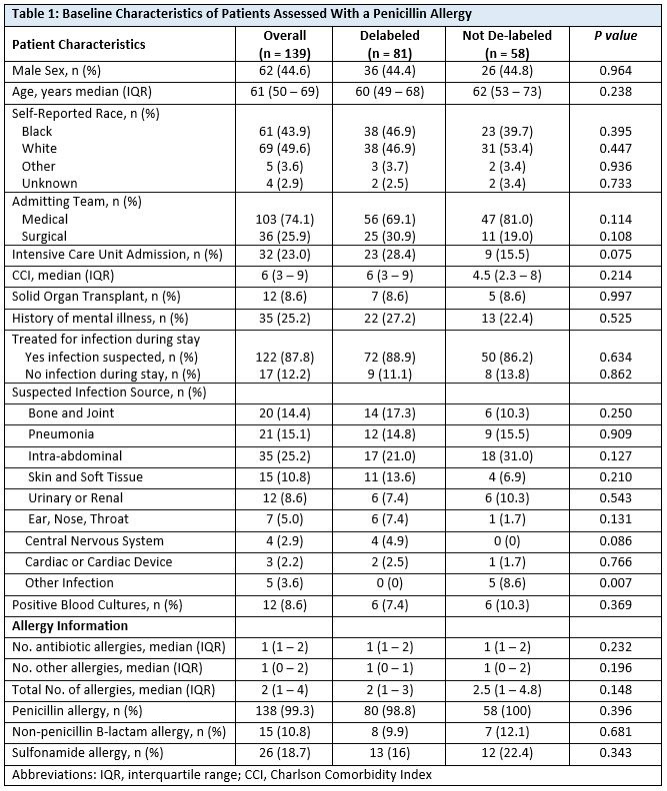

Patients were similar between groups on all baseline clinical and allergy characteristics except for more patients with infection classified as “other” in the non-delabeled group.

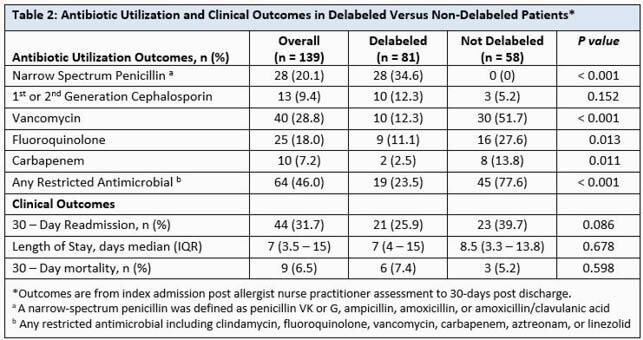

In the delabeled patients, we observed increased narrow-spectrum PCN use and decreased vancomycin, fluoroquinolone, carbapenem, and overall restricted antimicrobial use. Use of first and second generation cephalosporines was comparable between groups. Rates of 30-day readmission, LOS, and mortality were comparable.

**Conclusion:**

This QI effort between the departments of Allergy and ID to employ an ANP increased narrow spectrum antibiotic use and reduced use of restricted antimicrobials. Challenges included the part time position of the ANP unable to see every patient, reemergence of allergy in the chart, and clinical or other exclusions for delabeling (Fig 3).

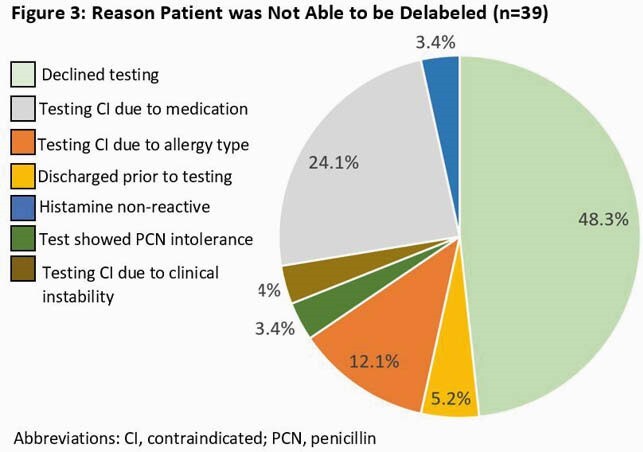

**Disclosures:**

**All Authors**: No reported disclosures

